# Topical glyceryl trinitrate for the treatment of tendinopathies: a systematic review

**DOI:** 10.1136/bjsports-2018-099552

**Published:** 2018-10-09

**Authors:** Dimitris Challoumas, Paul D Kirwan, Dmytro Borysov, Christopher Clifford, Michael McLean, Neal L Millar

**Affiliations:** 1 Institute of Infection, Immunity and Inflammation, College of Medicine, Veterinary and Life Sciences, University of Glasgow, Glasgow, UK; 2 School of Physiotherapy, Royal College of Surgeons in Ireland, Dublin, Ireland; 3 Physiotherapy Department, Connolly Hospital, Dublin, Ireland; 4 Physiotherapy Department, West Glasgow Acute Care Hospital, Glasgow, UK

**Keywords:** tendinopathy, tendinosis, tendon, treatment, overuse injury

## Abstract

**Objective:**

To produce a best evidence synthesis of the clinical effects of topical glyceryl trinitrate (GTN) in the treatment of tendinopathies.

**Design:**

A systematic review of published randomised controlled trials (RCTs) of the use of GTN in patients with tendinopathy.

**Data sources:**

MEDLINE, Embase, Scopus and CINAHL from database inception to January 2018.

**Methods:**

We examined RCTs comparing the effects of topical GTN with either placebo or other treatments on tendinopathy. Overall quality of each eligible study was determined based on a combined assessment of internal validity, external validity and precision. The level of evidence for each assessed parameter was rated based on the system by van Tulder *et al*.

**Results:**

A total of 10 eligible RCTs were identified including patients with tendinopathy of the rotator cuff (n=4), wrist extensors (n=3), Achilles (n=2) and patellar (n=1) tendons. For all tendinopathies, improvements in pain were significant when comparing GTN versus placebo in the short term (<8 weeks; poor evidence). Significant improvements in midterm outcomes for treatment with GTN versus placebo included the following: patient satisfaction (strong evidence); chances of being asymptomatic with activities of daily living (strong evidence); range of movement (moderate evidence); strength (moderate evidence); pain (at night and with activity; poor evidence) and local tenderness (poor evidence). Patients treated with topical GTN reported a higher incidence of headaches than those who received placebo (moderate evidence).

**Conclusions and relevance:**

Treatment of tendinopathies with topical GTN for up to 6 months appears to be superior to placebo and may therefore be a useful adjunct to the treating healthcare professions.

## Introduction

Overuse tendon injuries namely tendinopathies pose a significant clinical problem, particularly in musculoskeletal and sports-related medicine,^1^ accounting for up to 30% of general practice musculoskeletal consultations. The pathogenesis of tendinopathy is multifactorial and complex, and even though several theories have been suggested, the exact causative factors remain unknown.^2–7^ Our incomplete understanding of the mechanisms underpinning tendon pathophysiology continues to hamper the development of targeted therapies, which have been successful in other areas of musculoskeletal medicine.^8^ The most common exacerbating factor is thought to be overuse (particularly during sporting activities) causing repetitive microtrauma and consequent degeneration due to failure of the healing process.^2 6^ Manifestations range from mild pain and swelling to complete loss of function, and diagnosis is usually based on a thorough history and physical examination^4^; however imaging modalities such as ultrasound and MRI can be useful, especially for identifying tears.^9^ Tendinopathy appears to result from an imbalance between the protective/regenerative changes and the pathological responses that result from tendon overuse.^5 6^ The net result is tendon degeneration, weakness, tearing and pain.^10^

As the basic science of tendinopathy has evolved, so have the treatment options for these conditions. First-line treatment comprising several modalities ranging from relative rest and progressive loading to invasive pharmacological interventions continues to be the mainstay of treatment.^4^ Apart from loading which is widely recognised to be effective for the treatment of tendinopathies,^11^ the benefits of the remaining available therapies are equivocal, and treatment options are usually tried sequentially starting from the least noxious.^12^ The use of topical glyceryl nitrate (GTN), also known as nitroglycerin, for the management of tendinopathies was first reported by Berrazueta *et al*,^13^ who demonstrated successful treatment of acute rotator cuff tendinopathy with topical GTN. Due to the conflicting available evidence and its potential side effects, topical nitroglycerin is not currently licenced for the treatment of tendinopathies in the UK; however, it is sometimes used either on its own or alongside other treatment modalities based on evidence from several randomised controlled trials (RCTs).^14^

Nitric oxide (NO) is a free radical produced by a family of enzymes, the nitric oxide synthases (NOSs). Its involvement in tendon injury has been clearly demonstrated in the laboratory in several rodent studies, where all three NOS isoforms (b-NOS, e-NOS and i-NOS) were found to be upregulated both in acute and chronic tendon injuries,^15 16^ and tendon healing appeared to be reduced in rodents fed a competitive NOS inhibitor.^17^ Definitive conclusions on the exact role of NO in tendon healing are yet to be reached; however, experiments have shown that it likely enhances new tissue synthesis through its involvement in a number of processes, including local blood flow, host defence and collagen synthesis,^18^ all of which could potentially enhance the healing process of the injured tendon.

The limited existing evidence on the effectiveness of topical GTN on tendinopathy has reported conflicting results.^12^ In their Cochrane review assessing the effectiveness of topical GTN on rotator cuff tendinopathy specifically, Cumpston *et al* 14 concluded that there may be benefits on acute disease; however, evidence on chronic tendinopathy is insufficient. In the other relevant systematic review and meta-analysis, Gambito *et al* 19 analysed the effects of topical GTN on all tendinopathies and reported that there is strong evidence that GTN is effective in both relieving pain and increasing tendon strength. To our knowledge, no further relevant systematic reviews have been published since the study by Gambito *et al* 19 to examine the influence of subsequent RCTs on the outcomes in tendon disease.

The aim of this systematic review is to present the best available evidence on the effectiveness of topical GTN on tendinopathy and its side effects with a view to guiding future guidelines. After presentation of the findings of studies comparing topical GTN with placebo or alternative treatments, assessment of their quality and determination of the strength of available evidence, our specific objectives were to conclude on the effects of topical GTN in generic outcomes for each type of tendinopathy and all tendinopathies both in the short-term and midterm phases.

## Materials and methods

The present systematic review has been conducted and authored according to the Preferred Reporting Items for Systematic Reviews and Meta-Analyses (PRISMA)^20^ guidelines.

### Eligibility

Included studies were RCTs comparing at least one treatment group receiving topical GTN with a control group receiving either placebo or an alternative treatment. Studies with participants undergoing concurrent additional therapies were included only if both arms of the study received this additional treatment at the same frequency and intensity. Participants had to be over 18 years with a clinical diagnosis of tendinopathy with or without radiological signs. Duration of symptoms/signs was not a criterion, neither was length of treatment with, dosage and type of topical GTN used. Language criteria were not applied.

### Search strategy

A thorough literature search was conducted by two of the authors (DC and DB) independently via MEDLINE, Embase and Scopus in January 2018, with the following Boolean operators: ‘(GTN OR glyceryl trinitrate OR nitroglycerin) AND (tendinopathy OR tendinitis OR tendinosis OR rotator cuff OR supraspinatus OR shoulder OR patellar OR Achilles OR lateral epicondylosis OR lateral epicondylitis OR lateral epicondylopathy OR tennis elbow)’. Medical Subject Headinngs (MeSH)terms were not used to minimise the risk of missing relevant articles. Review articles were used to identify eligible articles that were missed at the initial search. Additionally, reference list screening and citation tracking in Google Scholar was performed for each relevant article.

### Screening

**Figure 1:**
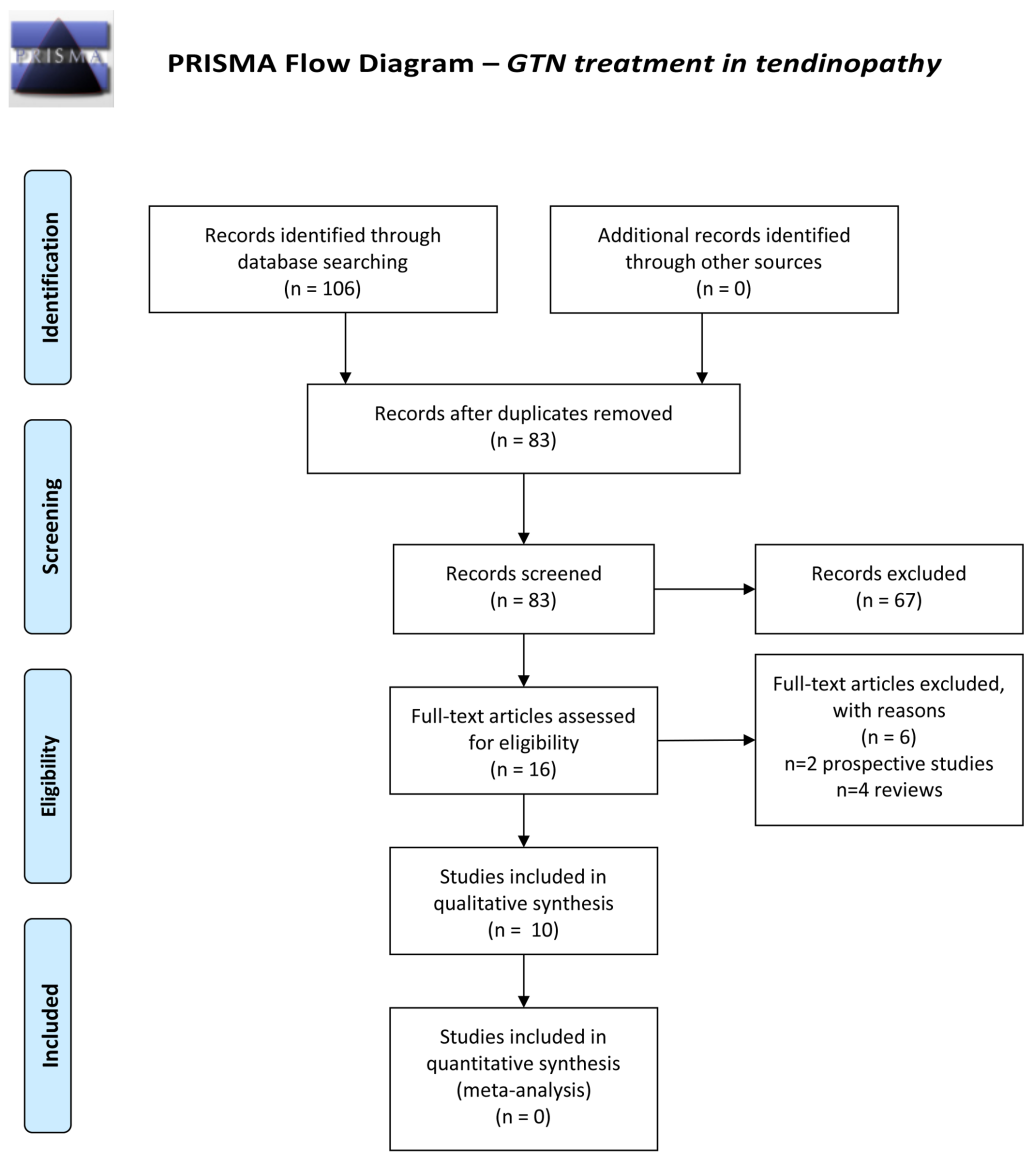
Preferred Reporting Items for Systematic Reviews and Meta-Analyses flow diagram of included studies.

From an initial total of 106 articles that were independently identified by two reviewers (DC and DB), after exclusion of duplicate and non-eligible articles, title and abstract screening and addition of missed studies identified by review articles, reference list screening and citation tracking, 10 studies were found to fulfil the inclusion and exclusion criteria. Figure 1 illustrates the article screening process according to PRISMA guidelines.^20^

### Quality assessment

A thorough quality assessment of the studies was conducted: all internal validity (freedom from bias), external validity (generalisability/applicability) and precision (reproducibility/freedom from random error) were assessed separately by two of the authors independently (DC and DB), and a third independent opinion (MM and CC) was sought where disagreements existed. Quality scales and resulting scores were not used as these usually combine aspects of study methodology with aspects of reporting; therefore, they are thought to be inappropriate for assessment of study quality.^21–23^ In addition, score cut-offs classifying studies of good or poor quality are usually not provided, and consequently, these are usually made up by the author of the review article, which can be highly variable. For internal validity, the ‘Cochrane Collaboration’s tool for assessing risk of bias in randomised trials’ was used, which includes six questions/criteria assessing the risk of six specific and one non-specific (‘other’) types of bias.^21^ As ‘other’ bias, our preset assessment criteria were: (A) adequate and appropriate inclusion and exclusion criteria, (B) differences between treatment and control groups at baseline (confounding) and (C) appropriateness of statistical tests deployed. External validity was assessed based on the population, age range and clinical relevance of interventions and outcome measures. For the assessment of precision the sample size, performance of statistical power calculation and p values that were used to define statistical significance were taken into account. In the Cochrane Collaboration’s tool, each item is classified as of ‘high’, ‘low’ or ‘unclear’ risk of bias. No total scores are given. As with the assessment of internal validity, external validity and precision of each study were separately rated as of ‘high’, ‘low’ or ‘unclear’ risk of bias.

Overall, studies were characterised as of ‘good’, ‘moderate’ or ‘poor’ quality based on a combined assessment of their internal validity, external validity and precision, which was again conducted by two of the authors independently (DC and DB) and the opinion of a third author (MM) was provided where the two judgements differed. The criteria used for overall quality assessment were as follows: ‘Good’ quality studies had ‘high’ risk of bias in <2 of the seven internal validity categories, external validity and precision; ‘Moderate’ quality studies had ‘high’ risk bias in 2 of the seven internal validity categories, external validity and precision; ‘Poor’ quality studies had ‘high’ risk of bias in >2 of the seven internal validity categories, external validity and precision.

### Data extraction: handling

Each of the eligible articles was initially read by the first author to gain familiarity, and subsequently each article was reread, and their key characteristics and findings were extracted and inserted in tables to facilitate analysis and presentation. For the presentation of results, outcomes were divided into short term and midterm, where follow-up findings at 2–8 weeks and 12–24 weeks were reported, respectively. Results for acute (symptoms less than 6 weeks) and chronic (symptoms more than 3 months) tendinopathy are also presented separately. For the classification of strength of evidence for each outcome reported, the rating system formulated by van Tulder *et al* 24–26 was used, which consists of four levels of evidence: strong evidence (level 1) is provided by generally consistent findings in multiple high-quality RCTs. Moderate evidence (level 2) is provided by generally consistent findings in one high-quality RCT and one or more low-quality RCTs or by generally consistent findings in multiple low-quality RCTs. Limited or conflicting evidence (level 3) is provided by only one RCT (either high or low quality) or by inconsistent findings in multiple RCTs. No evidence (level 4) is defined by the absence of RCTs. As our overall quality assessment included a ‘moderate’ quality category, we extended level 2 to evidence provided by generally consistent findings in high-quality RCT and 1 or more low-quality or moderate quality RCTs or multiple moderate quality RCTs. Two of the authors (DC and DB) jointly decided on the level of evidence for each outcome based on the aforementioned system without any disagreements. Results were considered to be significant when they were based on either moderate or strong evidence.

## Results

Table 1 and 2 and illustrate the characteristics of the included studies. A total of 10 eligible studies (figure 1) were identified with a total of n=584 participants (mean 58.4±38.1); of these, n=343 received GTN patches and n=241 control/no treatments (n=197 placebo patches, n=24 local corticosteroid injections, n=20 no treatment) (table 2). A total of n=317 participants in six studies^27–30^ received concurrent tendon rehabilitation (including eccentric strengthening exercises), n=154 in one study^31^ received concurrent stretching exercises and n=113 in three studies^13 32 33^ received no additional therapy (table 2). These additional therapies were thought to be administered at similar intensity and frequency in both treatment and placebo arms. Two of the studies assessed the effects of topical GTN on acute rotator cuff tendinopathy (n=68 participants) and the remaining eight on chronic tendinopathy (n=516; one study on patellar tendinopathy, two studies on rotator cuff tendinopathy, two studies on Achilles tendinopathy and three studies on lateral elbow tendinopathy). Dosages of topical GTN used varied from 0.72 mg/day – 5 mg/day (median 3.13 mg/day). Publication years ranged from 1996 to 2014. In the description of study findings, mean values of the most clinically relevant outcome measures of treatment and placebo groups at baseline and the longest follow-up time-point are presented where available. Mean values of visual analogue scale (VAS) for pain, which was the outcome measure used by most of the studies, at baseline and longest follow-up time-point for treatment and placebo groups are presented separately in table 3 along with the ‘treatment effect’ for pain, which we calculated using the following formula: (mean VAS of treatment group at follow-up – mean VAS of treatment group at baseline) – (mean VAS of placebo group at follow-up – mean VAS of placebo group at baseline). A negative value denoted improvement in pain with treatment compared with placebo and a positive value denoted worsening of pain. All values are presented at one decimal place.

**Table 1:** Methodological characteristics, inclusion and exclusion criteria, and follow-up completion rates of the included studies

Author	Study type	Randomisation method	Blinding method	Allocation concealment	Statistical power calculation	Baseline comparison	Inclusion criteria	Exclusion criteria	Follow-up completion
Berrazueta *et al* 13	Double-blind placebo-controlled RCT.	Not stated.	Not stated.	Not stated.	No.	Not done—greater pain and joint restriction in GTN group compared with control group as reported by Cumpston *et al.* 14	Acute supraspinatus tendintis (symptoms of <7 days) with tenderness and limitation of motion with increased pain with abduction.	Chronic shoulder pain, cervical spain/nerve root/brachial plexus lesions, cardiac/pulmonary/systemic disease, bone lesions or calcification at tendon insertion on X-ray, glaucoma, hypersensitivity to nitrates and NSAIDs.	100%
Pons *et al* 32	Double-blind placebo-controlled RCT.	Random number table.	Not stated.	Not stated.	No.	No difference.	Acute supraspinatus tendintis (symptoms of <6 weeks, pain on moving arm 60°–120° abduction, positive impingement test, Jobe, Gerbe or Pate tests, no response to 1-week NSAIDs treatment.	Adhesive capsulitis, biceps tendinitis, allergy/intolerance to GTN and those receiving GTN patches for heart disease.	100%
Steunebrink *et al* 35	Double-blind placebo-controlled RCT.	Sealed, coded envelopes; block randomisation (four participants per block).	Not stated.	Coordinated by independent physician; data after randomisation stored at secret location.	Yes; sample size adequate for 80% power.	Significantly higher VAS score during activities in control versus treatment group.	18–40 years, clinical diagnosis patellar tendinopathy (pain with activity or tenderness/thickening).	Symptoms of >24 months, VISA-P score >80, previous surgery or injection, previous eccentric programme in last 2 years, serious illness, pregnancy and contraindications to GTN.	82.5%
Giner-Pascual *et al* 33	Double-blind placebo-controlled RCT.	Allocation based on hour and date of clinic appointment.	Not stated	Not stated	No	GTN group older than control group; no baseline comparison of outcome measures.	>18 years, complete motor paraplegia, full-time wheelchair user, symptoms >3 months, diagnosis on MRI or US.	Tetraplegia, incomplete paraplegia, treatment with NO drugs and heart disease or hypotension.	91% (only 66.7% completed treatment)
Paoloni *et al* 29	Double-blind placebo-controlled RCT.	‘Coded randomisation’.	Active and placebo patches indistinguishable from each other.	Randomisation supervised by senior pharmacist.	Yes; sample size adequate for 90% power.	No difference.	>18 years, supraspinatus tendinopathy clinically (signs of impingement and pain in empty can position) and on MRI.	Symptoms of <3 months, pregnancy, previous surgery, previous shoulder dislocation, distal neurology, IHD and steroid injection last 3 months.	91%
Kane *et al* 30	Single-blinded, non-placebo-controlled RCT.	‘Sealed envelopes’.	Not stated.	Not stated.	No.	No difference.	Achilles tendinopathy confirmed on US and MRI.	Symptoms of <3 months, previous surgery or injection and contraindications to GTN.	90%
Paoloni *et al* 28	Double-blind placebo-controlled RCT.	Not stated.	Not stated.	Randomisation controlled by senior pharmacist.	Yes; sample size adequate for 90% power.	Significantly lower ankle plantar flexor mean total work in treatment than placebo group.	>18 years, diagnosis of non-insertional Achilles tendinopathy both clinically (insidious onset Achilles pain, tender nodule 2–6 cm proximal to calcaneal insertion) and radiologically (US scan with no tear).	Symptoms of <3 months, previous surgery, previous ankle dislocation, distal neurology, steroid injections in last 3 months and pregnancy.	89%
Ozden *et al* 34	Double-blind placebo-controlled RCT.	Not stated.	Not stated.	Not stated.	Yes; sample size adequate for 80% power.	No difference.	Tenderness and pain over lateral epicondyle, positive Mill’s sign, positive chair lift test, symptoms of >3 months and resisted wrist extension.	Surgery, effusion, radiculopathy, ulnar entrapment, fracture, infection, high ESR and injections.	100%
Paoloni *et al* 31	Double-blind, placebo-controlled RCT.	Stratified computer-generated randomisation.	Placebo and active patches identical in appearance.	Not stated.	Yes; sample size adequate for 80% power.	No difference in demographics; statistical analyses of outcome measures between groups not performed/stated.	18–70 years, symptoms of >3 months, VAS score >4/10 with provocative testing (ORI-TETS).	BMI >38, requirement of oral or topical analgesia, injection last 3 months, worker’s compensation cases, previous use of GTN, cardiac disease, pregnancy and previous surgery/fracture/dislocation.	88%
Paoloni *et al* 27	Double-blind placebo-controlled RCT.	‘Coded randomisation’.	Not stated.	Randomisation supervised by senior pharmacist.	Yes; sample size adequate for 90% power.	No difference in dropouts or outcome measures; statistical comparison of demographics between groups not reported.	>18 years.	Surgery, previous dislocation of wrist/elbow, injection in last 3 months and distal neurology.	86%

BMI, body mass index; ESR, erythrocyte sedimentation rate; GTN, glyceryl trinitrate; IHD, ischaemic heart disease; NO, nitric oxide; NSAIDs, non-steroidal anti-inflammatory drugs; ORI-TETS, Orthopaedic Research Institute Tennis Elbow Testing System; RCT, randomised controlled trial; US, ultrasound; VAS, visual analogue scale; VISA-P, Victorian Institute of Sports Assessment – Patella.

**Table 2:** Samples, characteristics of interventions and outcome measures of the included studies

Acute/ chronic tendinopathy	Tendon affected	Author	Sample, mean/median age, %F	Interventions	Treatment duration (follow-up)	Outcome measures
Acute	Rotator cuff	Berrazueta *et al* 13	n=20; mean 37 years (2062 years); 50%.	GTN patch 5 mg/day (n=10) or placebo patch (n=10).	3 days (24 hours, 48 hours, 15 days).	Pain: (A) VAS score. (B) Pain duration in last 24 hours (five-point scale). ROM: restriction of joint movement (five-point scale). Other: sleep hours (three-point scale). Treatment success: asymptomatic at 15 days.
Acute	Rotator cuff	Pons *et al* 32	n=48; mean 61 years; 69%.	GTN patch 5 mg/day (n=24) or local corticosteroid injection (n=24).	3 days; if no complete improvement, intervention was repeated up to 3 times at 15-day intervals (7 days, 22 days and 42 days).	Pain: at rest (changes in VAS score; described as three categories: decrease of >5 points=complete improvement, 3–5 points=partial improvement, <3 points= failure).
Chronic	Patellar	Steunebrink *et al* 35	n=33; median age 31.9 years in GTN group and 33.8 years in control group; NA.	GTN patch 1.25 mg/day+tendon rehab (n=16) or placebo patch+tendon rehab (n=17).	12 weeks (6 weeks, 12 weeks and 24 weeks).	Pain: VAS score during sports. Tendon specific: VISA-P questionnaire. Patient satisfaction.
Chronic	Rotator cuff	Giner-Pascual *et al* 33	n=45; mean 54.3 years GTN and 42.2 years control; 37.8%.	GTN patch 1.25 mg/day (n=33) or placebo patch (n=12).	6 months (6 months).	Pain: VAS score. ROM (degrees). Patient-group specific: (A) functional movement (SCIM). (B) Functional movement-induced pain (WUSPI).
Chronic	Rotator cuff	Paoloni *et al* 29	n=53; mean 51 years, median 52 years (25–79 years); 55%.	GTN patch 1.25 mg/day+tendon rehab (n=26) or placebo patch+tendon rehab (n=27).	24 weeks (2 weeks, 6 weeks, 12 weeks and 24 weeks).	Pain: at rest, at night and with activity (five-point scale: 0–4). Subacromial tenderness (four-point scale: 0–3). ROM: passive (degrees). Force (N). Tendon specific: Clinical impingement tests in IR and ER (positive or negative).
Chronic	Achilles	Kane *et al* 30	n=40; mean 40.5 years (22–68 years); %NA.	GTN patch 2.5 mg/day+tendon rehab (n=20) or tendon rehab only (n=20). Histology and immunohistochemistry of Achilles samples from surgery (control group n=3, GTN group n=4).	6 months (6 months)	Pain: AOS VAS for pain and disability. Other: (A) histology (neovascularisation, collagen synthesis and fibroblast activity). (B) Immunohistochemistry (NOS production).
Chronic	Achilles	Paoloni *et al* 28	n=65; median 49 years (24–77 years); 38.5%.	GTN patch 1.25 mg/day+tendon rehab (n=32) or placebo patch+tendon rehab (n=33).	24 weeks (2 weeks, 6 weeks, 12 weeks and 24 weeks).	Pain: (A) at rest, at night, with activity (five-point scale: 0–4). (B) After hop test (11-point scale: 0–10). Tenderness: four-point scale (0–3). Force: (A) plantar flexor peak force (N). (B) plantar flexor work (N).
Chronic	Wrist extensors	Ozden *et al* 34	n=40; mean 43.2 years (19–74 years), 30%.	GTN patch 1.25 mg/day+tendon rehab (n=20) or placebo patch+tendon rehab (n=20).	24 weeks (3 weeks and 6 months).	Pain: VAS score. Force: grip strength (N). Tendon specific: treatment outcomes as per Verhaar *et al.*
Chronic	Wrist extensors	Paoloni *et al* 31	n=154; NA years (NA years), NA%.	GTN patch 0.72 mg/day+stretching (n=41) GTN patch 1.44 mg/day+stretching (n=34), GTN patch 3.6 mg/day+stretching (n=44), placebo patch+stretching (n=35).	8 weeks (8 weeks)	Pain: at rest, with activity (VAS score). Force: (A) grip strength (kg) and (B) strength test using ORI-TETS (kg; tendon specific). Tendon specific: PRTEV. Other: SGAC in overall symptoms.
Chronic	Wrist extensors	Paoloni *et al* 27	n=86; median 46 years (30–74 years), 51%.	GTN patch 1.25 mg/day+tendon rehab (n=43) or placebo patch+tendon rehab (n=43).	24 weeks (2 weeks, 6 weeks, 12 weeks and 24 weeks).	Pain: at rest, at night, with activity (five-point scale: 0–4). Tenderness (four-point scale: none, mild, moderate and severe). Force: (A) Maudsley’s test (resisted third MCPJ extension force; N), (B) wrist extension peak force (chair pick up test - ORI-TETS; N; tendon specific) and (C) total work using ORI-TETS (N; tendon specific).

AOS, Ankle Osteoarthritis Scale; ER, external rotation; GTN, glyceryl trinitrate; IR, internal rotation; MCPJ, metacarpophalangeal joint; NOS, nitric oxide synthase; ns, not stated; ORI-TETS, Orthopaedic Research Institute Tennis Elbow Testing System; PRTEV, Patient-Rated Tennis Elbow Evaluation; ROM, range of movement; SCIM, spinal cord injury measurement; SGAC, Subjective Global Assessment of Change; VAS, visual analogue scale; VISA-P, Victorian Institute of Sports Assessment – Patella; WUSPI, Wheelchair Users Shoulder Pain Index.

**Table 3:** Mean values of VAS for pain where available

Tendinopathy	Study	VAS type (unspecified, at rest, at night, with activity)	VAS scale	GTN group		Placebo group		Treatment effect for pain (VAS 2 − VAS 1) – (VAS 4 − VAS 3)	P<0.05
				VAS baseline^42^	VAS longest follow-up^42^	VAS baseline (3)	VAS longest follow-up (4)		
Rotator cuff (acute)	Berrazueta *et al*	Unspecified	0–10	7.1	2	6	5.5	−4.6	Yes
Rotator cuff	Giner-Pascual *et al* 33	Unspecified	0–10	5.4	5.3	2.3	4.6	−2.4	Yes
Patellar	Steunebrink *et al* 35	With activity	0–10 (reverse)	4.1	6.6	5.8	7.8	−0.5*	No
Achilles	Kane *et al* 30	Unspecified	0–10	5.6	3.1	5.4	3	−0.1	No
Lateral epicondylitis	Paoloni *et al* 27	With activity	0–4	2.2	0.8	2.6	1.3	−0.1	No
	Paoloni *et al* 31P†	With activity	0–40‡	36	34	32	28.2	+1.8	No
	Paoloni *et al* 31P	At rest	0–40‡	36	35	32	29.4	+1.6	No
	Paoloni *et al* 31	At night	0–40‡	32	31.8	30	27.3	+2.5	No
	Ozden *et al* 34	Unspecified	0–10	8.1	0.7	8.8	4.9	−3.5	Yes

The statistical significance column (p<0.05) denotes whether there was a significant benefit in VAS for pain with topical GTN versus placebo.

*Due to the reverse VAS scale used (0=worst pain and 10=no pain), our subtraction was also reverse, that is, (VAS 1 – VAS 2) – (VAS 3 – VAS 4).

†In this study, only the mean VAS values of the highest strength GTN (3.6 mg/24 hours) group are presented for all pain with activity, at rest and at night; however, the lowest strength GTN group did have significantly less pain with activity at follow-up compared with placebo.

‡The VAS scale used is not stated in the article, but we assume based on the reported values and the previous article by the same group (were a VAS scale of 0–4 was used) that it is 0–40.

GTN, glyceryl trinitrate; VAS, visual analogue scale.

### Quality assessment

The quality assessment tools used are shown in table 4. Four studies were found to be of ‘poor’ overall quality, two of ‘moderate’ quality and four of ‘good’ quality.

**Table 4:** Assessment of internal validity, external validity, precision and overall quality of each study (see table 1 for criteria for overall study quality assessment)

Author	Internal validity (Cochrane’s Collaboration Tool for Assessing Risk of Bias)							External validity	Precision	Overall quality
	Selection bias		Performance bias	Detection bias	Attrition bias	Reporting bias	Other			
	Random sequence generation	Allocation concealment	Blinding of patients and staff	Blinding of outcome measures	Completeness of outcome data	Selective reporting				
Berrazueta *et al* 13	?	?	?	Low	Low	High	High	Low	High	Poor
Pons *et al* 32	Low	?	High	High	Low	High	High	High	High	Poor
Steunebrink *et al* 35	Low	Low	?	?	Low	High	High	Low	Low	Moderate
Giner-Pascual *et al* 33	Low	Low	?	?	High	High	High	High	High	Poor
Paoloni *et al* 29	?	Low	Low	Low	Low	High	Low	Low	Low	Good
Kane *et al* 30	?	?	High	?	Low	High	High	Low	High	Poor
Paoloni *et al* 28	?	Low	Low	Low	Low	Low	Low	Low	Low	Good
Ozden *et al* 34	?	?	?	Low	Low	High	Low	Low	Low	Good
Paoloni *et al*	Low	?	Low	Low	Low	High	High	Low	Low	Moderate
Paoloni *et al* 27	?	Low	?	Low	Low	High	?	Low	Low	Good

?, unclear risk of bias.

### Internal validity

#### Selection bias

All 10 studies were randomised. Three of the studies^13 28 34^ did not report any details about their randomisation method, while another three studies either stated ‘with coded randomisation’^27 29^ or ‘with sealed envelopes’^30^ without providing further details. Risk of bias with regard to allocation concealment was ‘low’ in four studies; the remaining six were classified as ‘unclear’ risk as details were not provided.

#### Performance bias

Two of the 10 studies were not double-blinded (‘high’ risk); one of them compared topical GTN with local corticosteroid injection^32^ and the other did not use placebo patches.^30^ Of the eight double-blinded studies, four failed to mention whether the active and placebo patches were indistinguishable from each other.^27 28 33 34^ (‘unclear’ risk).

#### Detection bias

Blinding of outcome measures was thought to be sufficient in seven studies (‘low’ risk) where the authors specifically state that the outcome assessors/examiners were blinded and/or did not participate in the assessment.^13 27–29 31 33 34^

#### Attrition bias

Reasons for dropouts/withdrawals of participants were adequately reported in all studies (‘low’ risk). Rate of follow-up completion was stated in all but three studies, wherein it was assumed to be 100% as suggested by their results tables.^13 32 34^. All studies had rates of follow-up completion greater than 80% (low risk; range 82.5%–100%). In the study by Giner-Pascual *et al*,^33^ only 66.7% completed treatment; however, some of the patients who dropped out participated in postintervention assessments resulting in follow-up completion of 91% (‘high’ risk).

#### Reporting bias

Reporting of results was appropriate and adequate in all but one study; Berrazueta *et al* 13 failed to provide statistical values for range of movement (ROM) and numerical or statistical values for hours of sleep. Despite adequate reporting of results, most studies were classified as ‘high’ risk of reporting bias due to selective reporting; they either failed to provide graphic illustration of significant results^13 27 29^ measured and reported a small number of outcomes^30 33–35^ or was terminated too early due to lack of significant findings.^31^

#### Other bias

Inclusion and exclusion criteria were thought to be adequate for all but one study, which only used as an inclusion criterion age more than 18 years and did not describe how the diagnosis of tendinopathy (lateral elbow tendinopathy) was made.^27^ Two studies did not exclude patients who had previous surgery or local corticosteroid injections,^13 33^ and the authors of one study state that ‘patients were excluded if they had any previous interventions such as local corticosteroid injection’, but it is not clear whether those who had previous surgery were excluded. Statistical comparison of treatment and control groups at baseline was thought to be inadequate in four studies: one did not perform a between-group comparison at baseline,^13^ two reported a comparison of demographics but not results of outcome measures^31 33^ and one presented a comparison of results of outcome measures but not demographics.^27^

#### External validity

General, non-specific populations were used in all studies but one, which included wheelchair user patients with complete motor paraplegia only.^33^ Age ranges of participants were wide enough to allow for good generalisability in all studies except for Steunebrink *et al*,^35^ where only young patients (18–40 years) were recruited. However, we do recognise that this age range is reflective of the population suffering from patellar tendinopathy (athletes in jumping sports). Clinically relevant assessment tools for pain were used in all studies apart from that by Pons *et al*,^32^ wherein only difference in pain was assessed with the use of an ‘analogue visual scale’, further details of which are not provided. Tendon-specific outcome measures were only used by five studies^26 28 30 33 36^ and functional questionnaires (Patient-Rated Tennis Elbow Evaluation (PRTEV) and Victorian Institute of Sports Assessment – Patella (VISA-P)) by two studies.^30 36^ No guidelines exist about the best formulation or dosage of topical GTN in clinical practice; therefore, all dosages used were considered clinically relevant.

#### Precision

Statistical power calculation prior to recruitment was performed in all but three studies,^13 30 33^ where performed sample sizes were adequate for a power of at least 80%. Levels of significance were set at p=0.05 in all studies; Paoloni *et al* 31 do not explicitly state their defined level of significance; however, this is assumed to be (at least) 0.05 as they consider their finding of p=0.04 significant.

### Included studies

#### Acute tendinopathy

##### Rotator cuff

Two published studies investigated the effects of topical GTN on acute rotator cuff tendinopathy. Berrazueta *et al* 13 found that at 24-hour 48-hour follow-up versus baseline: (A) the treatment group had significantly reduced intensity and duration of pain compared with placebo; (B) the treatment group displayed increased shoulder ROM in contrast to placebo; and (C) the treatment group had slightly improved hours of sleep compared with baseline versus placebo. When the effects of treatment were assessed 15 days following the 3-day intervention, all 10 patients in the treatment group were asymptomatic compared with 50% of patients in the control group. Two patients (20%) in the treatment group reported headache compared with 0 in the control group. In their study, Pons *et al* 32 repeated treatment up to three times at 15-day intervals when response was only partial, and pain was tested 7–10 days after treatment. In the corticosteroid group, ‘complete’ improvement was observed in 19 patients (79%), ‘partial’ (reduction by 3–5 points) in 3 patients (12%) and treatment failure (reduction by <3 points) in 2 patients (8%). In the GTN group, five patients (21%) had ‘complete’ improvement, 5 (21%) ‘partial’ improvement and treatment failed in 14 patients (58%). Headache was reported by 15 patients (62%) in the GTN group, of whom 8 (33%) had to discontinue treatment as a result and 0 in the corticosteroid group.

Overall, for acute rotator cuff tendinopathy only data on short-term outcomes are available from two studies of poor overall quality. Compared with placebo, topical GTN appears to be superior with respect to pain, ROM, hours of sleep and satisfaction (level 3 evidence). Compared with local corticosteroid injections, GTN appears to be less effective in improving pain (level 3 evidence).

#### Chronic tendinopathy

##### Rotator cuff

Two studies investigated the effects of topical GTN on chronic rotator cuff tendinopathy. In the study by Paoloni *et al*,^29^ at 2-week and 6-week follow-up, the only significant differences in the treatment group, compared with the control group, were an increased supraspinatus force and subscapularis force, respectively. At 12 weeks, the treatment group exhibited greater supraspinatus, subscapularis, adduction, internal rotation (IR) and external rotation (ER) force and less pain at rest and at night compared with control. At 24 weeks, the treatment group, compared with control, had: (A) less pain at rest, at night and with activity, (B) greater supraspinatus, subscapularis, ER, adduction and IR force, (C) greater ROM in abduction and IR and (D) less impingement in IR (Hawkins sign). Comparing treatment and control groups to baseline, at 24 weeks: (A) the former group had a significantly higher chance of being asymptomatic, (B) excellent improvement in pain was observed in 46% versus 24%, overall passive ROM increased by 24% versus 8%, (C) overall shoulder force increased by 29% versus 12% and (D) overall impingement signs decreased by 76% versus 43%, respectively. At week 24, the treatment group was significantly more likely to be asymptomatic with activities of daily living than the control group (46% vs 24%). The mean estimated effect size for all outcomes was 0.26. Headaches were reported by 58% patients in the treatment group and 33% in the control group and rashes by 12% and 4%, respectively.

In a subsequent study in wheelchair user patients with chronic rotator cuff tendinopathy, Giner-Pascual *et al* 33 reported favourable outcomes of topical GTN. Mean differences in WUSPI scores were also significant in the two groups between baseline and follow-up in favour of the GTN group. Comparing shoulder ROM at follow-up versus baseline, patients in the treatment group displayed significant increases in all directions as opposed to those in the control group, where a decrease was observed. The following side effects were reported in the treatment group: (A) headache 33% (vs 21% in control group) and (B) facial reddening 3%, tachycardia 3% and dizziness 3%.

Overall for chronic rotator cuff tendinopathy short-term outcomes from one study of good overall quality, showed the only significant difference between topical GTN and placebo was a greater subscapularis force in patients using GTN (level 3 evidence). Midterm outcomes were assessed by two studies, one of good^29^ and one of poor^33^ overall quality; significant results included less pain (level 3 evidence), higher ROM (level 2 evidence), higher overall force (level 3 evidence), higher satisfaction rates (level 3 evidence) and higher chances of being asymptomatic with activities of daily living (ADLs) (level 3 evidence) in patients using topical GTN versus placebo.

##### Patellar

The study by Steunebrink *et al* 35 is the only one investigating the effects of topical GTN on chronic patellar tendinopathy. At 24-week follow-up, both groups exhibited increases in the primary outcome (VISA-P score), but differences between them were non-significant (mean VISA-P in GTN group 63 at baseline and 75 at 24 weeks vs 67.8 and 80.7, respectively, in placebo group). Similarly, VAS scores and patient satisfaction rates (secondary outcomes) improved over time with no difference between the two groups. The only reported side effect was a rash in 19% patients in the treatment group.

In summary, no significant differences in short-term or midterm outcomes were identified in patients receiving topical GTN versus those receiving placebo patches by one study of moderate overall quality (level 3 evidence).

##### Achilles

Two RCTs assessed the effects of topical GTN on chronic Achilles tendinopathy. In the study by Kane *et al*,^30^ both groups had lower scores at the Ankle Osteoarthritis Scale (AOS) VAS scale for both pain and disability (mean AOS disability score in GTN group 3.5 at baseline and 2.25 at follow-up vs 3.95 and 2.15, respectively, in placebo group) at follow-up; however, no differences were detected between the two groups. In the treatment group, four patients (20%) had to discontinue patch application due to headaches, while no headaches were reported in the control group. Four patients in the treatment group and three in the control group went on to have surgical decompression as their symptoms had not improved after 6 months of treatment, and Achilles tendon samples were sent for histology and immunohistochemistry. No differences were found between the two groups in neovascularisation, fibroblast activity, collagen synthesis or production of e-NOS and i-NOS.

In the other RCT of chronic Achilles tendinopathy by Paoloni *et al*,^28^ compared with the placebo group, the treatment group had a significant decrease in: (A) Achilles tendon pain at night at 12 weeks; (B) pain with activity at 12 weeks and 24 weeks; (C) pain after the 10-hop test at 24 weeks; and (D) Achilles tendon tenderness at 12 weeks. Additionally, at 24 weeks, compared with baseline, the treatment group had a greater increase in plantar flexor mean total work than the placebo group. Finally, at 24 weeks, patients in the treatment group had a significantly higher chance of being asymptomatic with ADLs compared with those in the control group (78% vs 49%). Side effects were non-significant in treatment versus control groups: (A) headache 53% versus 45%, (B) rash 16% versus 12% and (C) increase in pre-existing tinnitus 3% versus 0%.

Overall, for chronic Achilles tendinopathy short-term outcomes comparing the use of topical GTN with placebo were reported by one study of good overall quality and no significant differences were detected in any of the outcomes (level 3 evidence). Midterm outcomes were reported by two studies: one of good^28^ and one of poor overall quality^30^; significant findings favouring GTN over placebo were reduced pain at night and with activity (level 3 evidence), local tenderness (level 3 evidence), increased force (level 3 evidence) and satisfaction (level 3 evidence) and higher chances of being asymptomatic with ADLs (level 3 evidence).

##### Lateral elbow tendinopathy

Three RCTs investigated the effects of topical GTN therapy on chronic lateral elbow tendinopathy (‘tennis elbow’). Paoloni *et al* 27 found that elbow pain with activity decreased significantly in both groups at all 2, 6, 12 and 24 weeks follow-up; however, a between-groups difference was only significant at 2 weeks, in favour of the treatment group. Similarly, both groups displayed significantly decreased lateral epicondyle tenderness at all follow-up stages compared with baseline; these decreases in the treatment group were significant compared with the placebo group only at weeks 6 and 12. Based on patient-reported outcomes, the treatment group was more likely to be asymptomatic with ADLs at week 24 compared with the control group (81% vs 60%, respectively). The mean estimated effect size for all treatment outcomes at week 24 was 0.12. Side effects were reported by treatment and placebo groups, respectively: headaches 63% versus 58%, rash 21% versus 9%, facial flushing 2% versus 0%, ipsilateral axillary sweating 2% versus 0% and apprehension 2% versus 0%.

Six years later, the same group^31^ conducted the largest RCT of its kind with 136 patients. According to the authors, the interventions were initially planned to be administered for 24 weeks; however, the study was abandoned at 8 weeks due to lack of significant results. Of all study outcomes (Subjective Global Assessment of Change, pain, PRTEV and strength), the only significant between-groups difference was a significant decrease in pain with activity at 8 weeks in the 0.72 mg/day GTN group compared with placebo. Mean pain-free grip strength (primary outcome) in the highest dosage GTN group was 23.1 at baseline and 30.5 at follow-up versus 22.9 and 27.4, respectively, in the placebo group. The authors did not report overall incidence of side effects; however, dropouts due to side effects were as follows: headache: n=2 (6%) in 1.44 mg/day GTN group and n=5 (11%) in 3.6 mg/day GTN group and dermatitis rash: n=1 (2%) in 3.6 mg/day GTN group.

In a recent study by Ozden *et al*,^34^ compared with baseline, both treatment and placebo groups had significant decreases in their pain VAS scores at both follow-up stages. The treatment group had significantly lower pain VAS scores compared with the placebo group at both 3 weeks and 6 months. Finally, at 6 months, 95% of patients in the treatment group reported excellent or good outcomes compared with 15% in the control group. Headaches were reported by 5% patients in the treatment group and 10% in the control group, of which no one had to abandon the study.

Overall for chronic lateral elbow tendinopathy, a total of three studies (two of good and one of moderate overall quality) compared short-term outcomes of topical GTN therapy versus placebo; significant differences favouring topical GTN include less pain (unspecified; level 3 evidence) and less pain with activity (level 2 evidence). Two studies (one of good and one of moderate overall quality) also described midterm outcomes; patients who received topical GTN had significantly less pain (level 3 evidence) and local tenderness (level 3 evidence) as well as greater force (level 3 evidence), satisfaction (level 2 evidence) and chances of being asymptomatic with ADLs (level 3 evidence) compared with those treated with placebo patches.

A summary of the results of the included studies on different patient-related outcomes is shown in table 5.

**Table 5:** Overall summary of results of different patient-related outcomes for chronic tendinopathy (table 5A and B) and acute tendinopathy (table 5C) separately

(A)													
Tendon affected	Author	Pain (unspecified)	Pain at rest	Pain at night	Pain with activity	Tenderness	ROM	Force	Satisfaction	Asymptomatic with ADLs	Function	Headache	Rash
Patellar	Steunebrink *et al* 35	–	–	–	↔	–	–	–	–	–	–	↔	↑
Overall patellar (evidence level)		– (4)	– (4)	– (4)	↔ (3)	– (4)	– (4)	– (4)	– (4)	– (4)	– (4)	–	–
Rotator cuff	Giner-Pascual *et al* 33	–	–	-–	–	–	–	–	–	–	–	↑	↔
	Paoloni *et al* 29	–	↔	↔	↔	↔	↔	↑	–	–	–	↑	↑
Overall rotator cuff (evidence level)		– (4)	↔ (3)	↔ (3)	↔ (3)	↔ (3)	↔ (3)	↑ (3)	– (4)	– (4)	– (4)	–	–
Achilles	Kane *et al* 30	–	–	–	–	–	–	–	–	–	–	↑	↔
	Paoloni *et al* 28	–	↔	↔	↔	↔	–	↔	–	–	–	↔	↔
Overall Achilles (evidence level)		– (4)	↔ (3)	↔ (3)	↔ (3)	↔ (3)	-– (4)	↔ (3)	– (4)	– (4)	– (4)	–	–
Wrist extensors	Ozden *et al* 34	↓	–	–	–	–	–	↔	–	–	–	↑	↔
	Paoloni *et al* 31	–	↔	–	↓	–	–	↔	↔ (SGAC)	–	↔ (PRTEV)	↑	↔
	Paoloni *et al* 27	–	↔	↔	↓	↓	–	↔	–	–	–	↔	↑
Overall wrist extensors (evidence level)		↓ (3)	↔^42^	↔ (3)	↓^42^	↓ (3)	- (4)	↔^42^	↔ (3)	– (4)	↔ (3)	–	–
Overall all tendinopathies (evidence)		↓ (3)	↔^42^	↔^42^	↔ (3)	↔ (3)	↔ (3)	↔^42^	↔ (3)	– (4)	↔ (3)	↑^42^	↔ (3)

Tendon affected	Author	Pain (unspecified)	Pain at rest	Pain at night	Pain with activity	Tenderness	ROM	Force	Satisfaction	Asymptomatic with ADLs	Function	Headache	Rash
(B)													
Patellar	Steunebrink *et al* 35	–	–	–	↔	–	–	–	↔	–	↔ (VISA-P)	↔	↑
Overall patellar (evidence level)		– (4)	– (4)	– (4)	↔ (3)	– (4)	– (4)	– (4)	↔ (3)	– (4)	↔ (3)	–	–
Rotator cuff	Giner-Pascual *et al* 33	↓	–	–	–	–	↑	–	–	–	–	↑	↔
	Paoloni *et al* 29	–	↔	↓	↓	↔	↑	↑	↑	↑	–	↑	↑
Overall rotator cuff (evidence level)		↓ (3)	↔ (3)	↓ (3)	↓ (3)	↔ (3)	↑^42^	↑ (3)	↑ (3)	↑ (3)	– (4)	–	–
Achilles	Kane *et al* 30	↔	–	–	–	–	–	–	–	–	–	↑	↔
	Paoloni *et al* 28	-	↔	↓	↓	↓	–	↑	↑	↑	–	↔	↔
Overall achilles (evidence level)		↔ (3)	↔ (3)	↓ (3)	↓ (3)	↓ (3)	- (4)	↑ (3)	↑ (3)	↑ (3)	- (4)		
Wrist extensors	Ozden *et al* 34	↓	–	–	–	–	–	↔	↑	–	–	↑	↔
	Paoloni *et al* 31	–	–	–	–	–	–	–	–	–	–	↑	↔
	Paoloni *et al* 27	-	↔	↔	↔	↓	–	↑	↑	↑	–	↔	↑
Overall wrist extensor (evidence level)		↓ (3)	↔ (3)	↔ (3)	↔ (3)	↓ (3)	- (4)	↑ (3)	↑^42^	↑ (3)	– (4)		
Overall all tendinopathies (evidence level)		↓^42^	↔^42^	↓ (3)	↓ (3)	↓ (3)	↑^42^	↑^42^	↑^42^	↑^42^	↔ (3)	↑^42^	↔ (3)

(C)													
Tendon affected	Author	Pain (unspecified)	Pain at rest	Pain duration	ROM	Hours of sleep	Satisfaction	Headache	Rash				
Rotator cuff	Berrazueta *et al* 13	↓	–	↓	↑	↑	↑	↑	↔				
	Pons *et al* 32	–	↑	–	–	–	–	↑	↔				
Overall rotator cuff (evidence level)		↓ (3)	↑ (3)	↓ (3)	↑ (3)	↑ (3)	↑ (3)	↑ (3)	↔ (3)				

Short-term (0–8 weeks) and midterm (12–24 weeks) outcomes of chronic tendinopathy are presented separately in table 5A and B, respectively. Level of evidence is provided in brackets for each overall outcome separately.

ADLs, activities of daily living; ROM, range of movement.

## Discussion

The results of this systematic review provide good evidence for the effectiveness of topical GTN for the treatment of tendinopathies compared with placebo in the short and intermediate term (<6 months). Treatment with topical GTN for 12–24 weeks was associated with increased ROM in chronic rotator cuff disease (moderate strength evidence), and for all chronic tendinopathies topical GTN had positive effects on satisfaction (strong evidence), chances of being asymptomatic with ADLs (strong evidence), unspecified pain (moderate strength evidence), ROM (moderate strength evidence) and tendon force (moderate strength evidence). Pain at rest was unaffected by treatment with topical GTN for the same period for all chronic tendinopathies (strong evidence); however, it should be remembered that tendons are rarely painful at rest but typically when loaded, and thus this finding may not be clinically relevant. Overall effects of topical GTN on pain at night, pain with activity and local tenderness may also be beneficial; however, this is only based on poor strength evidence. Effects of treatment for shorter periods (<8 weeks) seem to be less pronounced. Equally, conclusions on the effects of topical GTN on acute tendinopathies could not be drawn due to the lack of high-quality evidence; however, there may be benefits in pain, ROM and sleep in patients with acute rotator cuff disease based on a single study with high risk of bias (poor strength evidence). Finally, with regard to side effects, topical GTN seems to be associated with a higher incidence of headaches (moderate strength evidence) while its effect on rashes seemed to be non-significant; however, this was only based on evidence of poor strength.

Long-term effects of topical GTN in tendinopathy have only been assessed by two prospective studies, which were not included in the present review as they did not fulfil the eligibility criteria^37 38^ (non-RCTs). Paoloni *et al* 37 followed up 52 of the participants (80%) of their previous study^28^ that compared the effects of topical GTN and placebo patches on Achilles tendinopathy 3 years later. Additionally, the authors included an assessment using the Victorian Institute of Sports Assessment – Achilles (VISA-A) scale; however, they did not perform measurements of plantar flexor peak force and plantar flexor work. Compared with the control group (n=28), the GTN group (n=24) had significantly decreased tenderness, a higher mean VISA-A score and a greater chance of being asymptomatic with ADLs (88% vs 67%). All other outcome measures showed a non-significant trend towards improvement. The estimated mean effect size of all outcome measures at 3 years for topical GTN was 0.21. Similarly, McCallum *et al* 38 followed up a total of 58 participants (67%) from their previous study^27^ on lateral elbow tendinopathy 5 years after its completion and performed the same assessments. The authors found that the improvements in all outcome measures, which were reported at 6 months (pain with activity, local tenderness, wrist extensor peak force and total work), were sustained at 5 years; however, no significant differences were detected between the treatment and control groups. This suggests that this improvement in both groups was most likely a direct result of the tendon rehabilitation that all participants received and/or time. The most important limitation of these prospective studies was that certain patients received additional treatments (more GTN patches, extracorpeal shock wave therapy (ESWT), acupuncture, herbal therapies and surgery), which were not adjusted for and could have confounded the results.

The findings of previously published reviews assessing the effects of topical GTN on tendinopathy are partly in agreement with our results. A systematic review and meta-analysis of seven RCTs on all types of tendinopathy^19^ concluded that there is strong evidence that topical GTN relieves pain during activity and increases tendon strength. We have now included another three studies that were published in the interim and used a different approach in assessing the quality of studies; in contrast to Gambito *et al*,^19^ we refrained from using quality scales and resulting scores as these usually assess study reporting in addition to study methodology; therefore, they are thought to be inappropriate for assessment of study quality. A recent systematic review and meta-analysis of 22 RCTs^39^ comparing the effects of all non-surgical treatments (including topical GTN) to no treatment in lateral elbow tendinopathy reported no significant intermediate to long-term benefits of non-surgical treatments over observation only or placebo. Equally, another systematic review of 12 RCTs looking at the effects of pharmacological interventions for Achilles tendinopathy^12^ concluded that there is lack of significant evidence to support the use of any of the therapies studied (topical GTN and injections of platelet rich plasma (PRP), autologous blood, polidocanol, corticosteroid, aprotinin, prolotherapy and fibroblasts) as they provided no significant benefits in terms of pain, disability, quality of life or histological changes compared with no treatment.

Finally, it is important given our incomplete understanding of the mechanisms underpinning tendon pathophysiology to consider the mechanism of action of GTN in the setting of tendon disease. Following injury to a tendon, NO is produced by all three isoforms of NOS^17^: NOS activity is upregulated in tendinopathy.^15^ In an exercise-overuse model of tendon degeneration, *i-NOS*, *e-NOS* and *b-NOS* mRNAs were overexpressed in the supraspinatus tendon of rats subjected to treadmill running for 14 days^40^. Expression of all isoforms was confirmed in human tendon disease from biopsy samples taken during shoulder surgery,^16^ while cultured human tenocytes exposed to exogenous NO increased total collagen synthesis.^41^ This supports the notion that NO enhances extracellular matrix synthesis and results in injured tendons having better material and mechanical properties. Despite these useful ‘preclinical’ findings, little follow-up work has been done to elucidate the optimum method of delivery of NO to tendons in an attempt to realise clinical efficacy. Thus, further work is required to move pastsimple ‘patch’ therapy, which suffers issues with drug delivery dosages, and ongoing trialss may well help answer these queries (ClinicalTrials.gov Identifier: NCT02499484).

Despite the rigour of our review with respect to identifying all the available evidence and the quality assessment of the included studies, we do recognise its limitations. First, due to the small number of eligible studies, our results on most outcomes had a poor level of evidence, especially for specific types of tendinopathies. Additionally, the different dosages of topical GTN and outcome measures used resulted in lack of homogeneity, which made the conduction of a meta-analysis impossible. Finally, the effects of the concurrent physiotherapy (eccentric and stretching exercises) that most participants received might have affected the results, even though, where used, both treatment arms were instructed to perform the same exercises at the same frequency and intensity, the actual frequency, intensity and correct performance of the exercises were not assessed.

## Conclusion

The results of this review provide good evidence for the effectiveness of GTN in the short and intermediate term treatment of tendinopathies (<6 months). GTN treatment is thus a good example that translational tendinopathy (laboratory bench to patient) can provide pharmacological adjuncts to aid the practising healthcare professional in addition to loading regimes. Importantly, other than headaches and occasionally rashes, topical GTN is a safe and practical treatment modality with very low costs both for the patient and the healthcare system. Therefore, the use of topical GTN should be considered for all chronic tendinopathies as an adjunct to loading programmes that fail to produce satisfactory resolution of symptoms. However, physicians should alert patients that large, well-designed RCTs and prospective cohort studies are warranted to provide convincing evidence on the effects of topical GTN in both acute and chronic tendinopathy, especially its long-term outcomes.

**What is already known**

- Twenty years since concept of glyceryl trinitrate (GTN) therapy in tendinopathy with still no clear guidance/evidence of efficacy.

New findings

- Ten eligible randomised controlled trials in all tendinopathies reveal improved midterm (up to 6 months) improvements in pain, strength and patient satisfaction.
- Main adverse event is headaches in up to one in five patients.
- Topical GTN is useful for all chronic tendinopathies as an adjunct to loading programmes that fail to produce satisfactory resolution of symptoms.
